# Estimating flexibility preferences to resolve temporal scheduling conflicts in activity-based modelling

**DOI:** 10.1007/s11116-022-10330-8

**Published:** 2022-10-16

**Authors:** Patrick Manser, Tom Haering, Tim Hillel, Janody Pougala, Rico Krueger, Michel Bierlaire

**Affiliations:** 1 Swiss Federal Railways (SBB), Hilfikerstrasse 1, 3000 Bern 65, Switzerland; 2 École Polytechnique Fédérale de Lausanne (EPFL), Transport and Mobility Laboratory (TRANSP-OR), Station 18, 1015 Lausanne, Switzerland; 3 University College London (UCL), London, UK; 4 Department of Technology, Management and Economics, Technical University of Denmark (DTU), 2800 Kongens Lyngby, Denmark

**Keywords:** Activity-based model, Discrete choice, Mathematical optimisation, Maximum likelihood estimation, Mixed-integer linear program

## Abstract

This paper presents a novel activity-based demand model that combines an optimisation framework for continuous temporal scheduling decisions (i.e. activity timings and durations) with traditional discrete choice models for non-temporal choice dimensions (i.e. activity participation, number and type of tours, and destinations). The central idea of our approach is that individuals resolve temporal scheduling conflicts that arise from overlapping activities, e.g. needing to work and desiring to shop at the same time, in order to maximise their daily utility. Flexibility parameters capture behavioural preferences that penalise deviations from desired timings. This framework has three advantages over existing activity-based modelling approaches: (i) the time conflicts between different temporal scheduling decisions including the activity sequence are treated jointly; (ii) flexibility parameters follow a utility maximisation approach; and (iii) the framework can be used to estimate and simulate a city-scale case study in reasonable time. We introduce an estimation routine that allows flexibility parameters to be estimated using real-world observations as well as a simulation routine to efficiently resolve temporal conflicts using an optimisation model. The framework is applied to the full-time workers of a synthetic population for the city of Lausanne, Switzerland. We validate the model results against reported schedules. The results demonstrate the capabilities of our approach to reproduce empirical observations in a real-world case study.

## Introduction

Planning for future transport infrastructure and efficient service concepts in light of possible changes in population (e.g. demographic shifts), policy or technology relies on quantitative forecasts of travel demand. To that end, simulation models which aim to closely represent mobility behaviour are applied. Current state-of-the-art transport models adopt an activity-based (i.e. microscopic) approach (Castiglione et al. 2015). Activity-based transport models treat mobility as individual decisions across many interwoven choice dimensions. These models afford a high-resolution representation of travel behaviour as they simulate each traveller as an autonomous decision-making unit and consider full consistency in time and space over a time period (e.g. a 24h-day) for each individual (Rasouli and Timmermans 2014). In most activity-based models, the different choice dimensions – e.g. activity participation, destinations, activity duration or start times – are simulated sequentially (Castiglione et al. 2015; Davidson et al. 2011). Different modelling approaches can be used for each choice dimension. For example, activity participation as well as destination and mode decisions are well suited to discrete choice models. Particularly destination and mode choice models are demonstrated to be well calibrated in various applications (Hörl et al. 2019; Scherr et al. 2020a). In comparison, the temporal choice dimension (i.e. activity duration and timings) remains under-explored in the literature. These decisions are either modelled based on rule-based and data-driven approaches (Scherr et al. 2020a) or based on simplified behavioural assumptions, e.g. with a transformation of the continuous timings into discrete intervals (Vovsha et al. 2005).

Sequential and rule-based or data-driven approaches for generating activity-based schedules are found wanting since they only partially consider behavioural preferences and interactions between choice dimensions. To date, behavioural preferences for the temporal scheduling decisions have been modelled either as a discrete variable (Vovsha et al. 2005; Castiglione et al. 2015) or as a continuous variable with heuristic and simplified flexibility parameters (Javanmardi et al. 2016; Esztergár-Kiss et al. 2020). A framework to capture the interactions between choice dimensions was introduced by Pougala et al. (2022). However, the framework is only applied to a small sample of individuals and the applicability in a large-scale context is not demonstrated. Importantly, the heuristic flexibility parameters are selected heuristically. In this way, the preferences are specified by the modeller and not by statistical estimation routines.

This work applies a novel activity-based model which is able to resolve scheduling conflicts in the temporal dimension based on estimated individual flexibility parameters for a real-world large-scale scenario. Non-temporal choice dimensions (i.e. activity participation, bundling into tours, and destinations) are simulated using the existing discrete choice models from Scherr et al. (2020a). The model aims to resolve activity-scheduling conflicts that arise from overlapping activities for each individual. In the case of a scheduling conflict, the flexibility parameters capture the behavioural preferences to shift timings of certain activity types compared to others. The flexibility parameters are quantified using maximum likelihood estimation. For the simulation of the conflict resolution, we build on the theoretical mathematical model introduced by Pougala et al. (2022). The framework allows to simulate desired choice dimensions simultaneously using a mixed-integer linear program (MILP), which has two major advantages: (i) it allows for interactions between multiple choice dimensions; and (ii) conflicts in time and space among different activities are resolved based on behavioural preferences. In this paper, we introduce another contribution to the framework by providing a computationally efficient implementation to demonstrate the application capabilities to a synthetic population for a city-scale case-study, with a detailed validation against empirically available observations. The output of the framework is a realistic daily schedule containing a sequence of activities for each simulated individual. Each activity in this schedule has a certain type, start time, duration, location as well as a mode to travel to the following activity. The schedules are an essential input for a traffic assignment – e.g. using the software MATSim (Horni et al. 2016) – which can be used to derive network loads to support decisions about future transport infrastructure investments.

The remainder of the paper is structured as follows: first, a brief review of recent advances in activity-based demand modelling is provided. Next, the framework is introduced, including the parameter estimation routine and the MILP. This framework is then applied to the group of full-time workers of a synthetic population in the case study for the city of Lausanne, Switzerland. Finally, the results are presented, validated against empirical data from a survey and discussed.

## Background

Early activity-based approaches to travel demand modelling were proposed in the 1990s (Axhausen and Gärling 1992). The main motivation driving the transition from traditional aggregated models to activity-based models is the lack of behavioural realism in the traditional approach, which does not allow for forecasting new policies such as congesting pricing, teleworking and ride-sharing incentives (Rasouli and Timmermans 2014). For a more detailed overview of the development of the activity-based approach, we direct the reader to the reviews provided by Bowman (2009) and Castiglione et al. (2015).

The first microscopic activity-based models were developed for several Northern American cities (Bowman and Ben-Akiva 2001; Vovsha et al. 2005; Bhat et al. 2004). They follow an econometric, utility-maximising approach to simulate the choice behaviours of households and individuals. Typically, the activity schedules are built using a set of discrete choice models for mobility tool ownership, tour bundling, activity selection, mode choice, and location choice. Multinomial and nested logit models are the most commonly used model forms in practice to represent the interactions of the various dimensions and to link the separate model steps. As Bhat et al. (2004) highlight, several important structural issues are not addressed within these early models, one of them being the relation of the time-of-day decision to the mode and destination choices.

Other early implementations of activity-based models use a rule-based approach. For example, the ALBATROSS framework (Arentze and Timmermans 2004) uses decision trees to represent choice heuristics of individuals and derives these heuristics from travel data. Other rule-based frameworks that use decision trees for the scheduling procedure are FEATHERS (Bellemans et al. 2010) and TASHA (Miller and Roorda 2003; Roorda et al. 2008). As stated in Auld et al. (2009), the issue with rule-based models is that they cannot predict modification choices, i.e. if an activity is modified, is it moved or shortened and by how much.

In Europe, the development of activity-based models was arguably driven by the increasingly popular agent-based transport simulation software MATSim (Horni et al. 2016). Most activity-based models are developed to generate schedules which are then fed into the dynamic traffic assignment of MATSim. The software MATSim itself provides a well-elaborated network simulation and route searching algorithms. However, the standard version of MATSim does not provide the functionality to generate activity-based demand including the choice dimensions of activity participation, sequence, locations, and initial timings. For this purpose, Ziemke et al. (2015) use the econometric framework CEMDAP (Bhat et al. 2004) to couple an existing activity-based model with MATSim. They take the original discrete choice parameters as estimated for Los Angeles, and apply them to a synthetic population of Berlin. Hilgert et al. (2017) propose the framework mobiTopp that covers the time period of one week. It is also inspired by the approach of Bowman and Ben-Akiva (2001) and applies a set of discrete choice models. Activity durations and start times are simulated as combination of discrete choice of an aggregated category and weighted random draws within the chosen category. The resulting activity-based schedules of the mobiTopp model are then integrated into MATSim (Briem et al. 2019). A very similar approach is presented in Scherr et al. (2020a). They show a comprehensively validated microscopic model called SIMBA MOBi in which agents react to transport supply across all mobility choices. For the choices in the temporal dimension, it applies a rule-based approach that is based on weighted random draws from empirical distributions. Moeckel et al. (2020) propose a framework called MITO containing a simplified activity-based model. Destination choice is influenced by a travel time budget for every household, i.e. people who spent a lot of time commuting are less likely to do much other travel. A recent study from Hörl and Balac (2021) introduces a standardised process for generating activity-based travel demand based on open data and open software that is fully replicable by any user. Their approach is predominantly data-driven and does not focus on behavioural parameters. Drchal et al. (2019) implement another fully data-driven approach.

A newer generation of activity-based model tries to solve the scheduling problem with techniques like Hidden Markov Models or Bayesian Networks. Both Liu et al. (2015) and Saadi et al. (2016) introduce a Hidden Markov Model. The advantage of this method is that it considers trends of their activity sequencing from a temporal perspective. However, Saadi et al. (2016) point out that the approach presents a limitation at the temporal dimension. Joubert and De Waal (2020) present a Bayesian Network approach and highlight the benefits of a behaviourally rich travel demand model which allows for causal interpretation. This method can also account for temporal variables like activity duration.

There have been few attempts to combine activity-based modelling and mathematical optimisation techniques reported in the literature. An early study by Recker (2001) presents a theoretical mathematical formulation targeting to facilitate the practicality of activity-based modelling approaches. It unifies the complex interactions among the scheduling conflicts solved by households in performing their daily activities, while preserving the utility-maximising principles. Building from this work, Recker et al. (2008) introduce an estimation procedure for this optimisation framework. They estimate the relative importance of factors associated with spatial and temporal interactions among the activities in a schedule, however they conclude that the formulation for the time sequence is rather simplistic. Another framework called ADAPTS is demonstrated by Javanmardi et al. (2016), which implements a flexible non-linear optimisation model. The model is applied to a synthetic population for the Chicago region. The objective function aims to minimise the amount of changes in timing and duration of involved activities in a conflict situation. The weights used to account for individual activity preferences are constant weights for all activity types. Rizopoulos and Esztergár-Kiss (2020) develop an optimisation model for the interaction of activity scheduling and charging electric vehicles. For this purpose, the authors use a Genetic Algorithm that considers temporal flexibility which is defined based on heuristic rules and priority labels per activity. Esztergár-Kiss et al. (2020) suggest an activity-based schedule optimisation method that includes temporal and spatial flexibility of the activities using a modified version of the Traveling Salesman Problem with Time Window constraints. Similar to Rizopoulos and Esztergár-Kiss (2020), they define heuristic flexibility priorities. Also, they focus on the travel episodes with a rather simple utility specification. Ballis and Dimitriou (2020) aim to convert multi-period and purpose-dependant origin-destination matrices into sets of activity schedules. They show a comprehensively validated framework that is mainly established on advanced graph-theoretical and combinatorial optimisation concepts. However, they only use simplified activity types and the activity schedules are not accompanied by socio-demographic information.

Many of the discussed issues are addressed by the activity-scheduling framework proposed by Pougala et al. (2022). They provide a theoretical framework that combines multiple choice dimensions (activity participation, location, start time, duration and mode choice) into a single optimisation problem and that captures the complex trade-offs between scheduling decisions for multiple activities. The framework is based on the behavioural principle that individuals maximise their overall schedule utility according to their preferences and constraints for performing desired activities during one day. It represents all choices in the time dimension as continuous variables. The utility formulation is modular and preferences can be specified for each individual activity. The optimisation model is implemented as a MILP that can easily be extended by additional custom constraints. In their work, Pougala et al. (2022) take flexibility parameters from the literature without statistical estimation. Also, they demonstrate the framework for small samples of individuals only and do not proof the applicability in the large-scale context with a detailed validation against empirical observations.

The current work fills the mentioned gaps in the literature by focusing on resolving scheduling conflicts in the temporal dimension, on estimating the individual flexibility parameters and on computational efficiency to scale the model to a real-world large-scale simulation. Stated succinctly, the contributions of our work are as follows:

- *Flexibility parameters*: An estimation routine is demonstrated that considers the temporal decision within the scheduling problem as a whole. In contrast to existing approaches (Bowman and Ben-Akiva 2001; Bhat et al. 2004; Hilgert et al. 2017; Scherr et al. 2020a) which consider trip- or tour-based parameters, this approach estimates schedule-based parameters (total daily utility). Our formulation considers time as a continuous variable and the resulting flexibility parameters are based on behavioural principles. In the literature, time has either been modelled as a discrete variable in combination with utility-based principles (Bowman and Ben-Akiva 2001; Castiglione et al. 2015) or as a continuous variable in combination with heuristic flexibility parameters (Javanmardi et al. 2016; Esztergár-Kiss et al. 2020; Pougala et al. 2022).
- *Real-world Application*: The implemented approach of coupling traditional discrete choice models with an optimisation model to reduce the number of decision variables targets to proof the applicability of the framework in a real-world large-scale case study, which is not done in Pougala et al. (2022). This work applies the scheduling model to all 50’000 full-time workers of a synthetic population of Lausanne, Switzerland. The size is comparable to studies in the literature, which mostly simulate a certain percentage (e.g. 10 %) of the synthetic population for bigger cities (Ziemke et al. 2015; Briem et al. 2019).

## Methodology

**Fig. 1:**
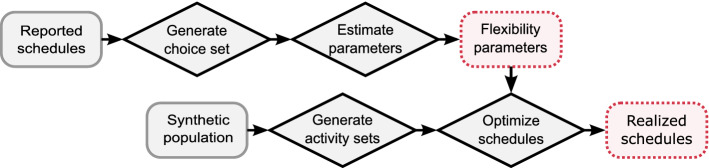
The two streams of the scheduling framework: estimation (top row) and simulation (bottom row)

The presented work implements an approach that makes use of existing traditional discrete choice models and combines them with an adapted version of the mathematical model as proposed by Pougala et al. (2022). This approach aims to reduce the complexity of the problem and puts focus on parameter estimation and real-world application. To calibrate the individual flexibility parameters for a case study, we introduce an estimation routine that quantifies behavioural preferences based on empirically reported schedules in the Swiss mobility and transport microcensus (MTMC) (BfS and ARE, 2017). The choice set for the estimation routine is generated using existing sequential discrete choice models (Scherr et al. 2020a). Based on the flexibility parameters, an optimisation model solves all temporal scheduling decisions (start times and durations of each activity) simultaneously while considering travel times. The non-temporal elements of the scheduling problem (i.e. activity participation, number and type of tours, and considered destinations) are simulated using the traditional discrete choice models as introduced by Scherr et al. (2020a) outside of the optimisation. They are fed as static activity sets into the optimisation framework. The proposed framework can be summarised with the two following streams, as illustrated in Fig. 1:

1. *Parameter estimation:* The first stream (top row in Fig. 1) aims to estimate individual preferences for a specific person group based on schedules reported in the MTMC (BfS and ARE 2017). In this work, we focus on the group of full-time workers. Individual preferences are flexibility parameters that express the loss of utility when either deviating from a desired starting time or duration for each given activity type. The procedure is described in more detail in Section "Estimation of individual flexibility preferences".
2. *Schedule simulation:* The second stream (bottom row) optimises the utility of the daily activity schedule for each individual in the synthetic population. The methodology to generate a synthetic population is provided in Bodenmann et al. (2019) and goes beyond the scope of this paper. Each individual is assumed to choose an activity set that they are going to perform during a 24h-period. Based on the estimated flexibility parameters, the optimisation approach then resolves time conflicts that arise from overlapping desired timings in the activity set. More insights into the methodology are given in Section "Schedule optimisation".

### General definitions

The following definitions are important in the context of this work:

- *Activity*: Each $a \in {\mathcal {A}}$ is defined by a specific type, a desired start time $x^{*}$, a desired duration $\tau ^{*}$, and a set considered locations ${\mathcal {L}}$. Also, each activity is assigned to a tour type and an activity can be defined to be a sub-tour activity.
- *Activity set*: An activity set ${\mathcal {A}}$ contains all activities that an individual needs or wishes to perform during a time period (e.g. a 24-hour day) in no sequential order.
  It can contain multiple instances of activities of the same type (e.g. two leisure activities). An example for an activity set is ${\mathcal {A}} = \{dawn, ~dusk, ~home, ~work, ~leisure\_\textit{1}, ~leisure\_\textit{2} \}$. In this case, $leisure\_\textit{1}$ and $leisure\_\textit{2}$ are both leisure activities, but may have different desired start times, desired durations and considered location sets. We differ between three subsets of ${\mathcal {A}}$:
- *Home activity set*: The set ${\mathcal {H}} \subseteq {\mathcal {A}}$ contains the number of home activities an individual is going to perform. It includes activity types *home*, *dusk* and *dawn*. ${\mathcal {H}}$ must contain exactly one dawn and one dusk activity, whereas the number home activities is not constraint. We assume that each person wants to spend a fraction of their daily time budget at home. Therefore, we define $\tau ^{*}_{\mathcal {H}}$ as the desired *home time budget*, which is the sum of the durations of all in-home activities: $\sum _{a \in {\mathcal {H}}} \tau _a = \tau _{\mathcal {H}}$. Each $h \in {\mathcal {H}}$ take place at the same location ($|{\mathcal {L}}|=1$) as defined in the synthetic population data.
- *Primary activity set*: The set ${\mathcal {P}} \subset {\mathcal {A}}$ includes activity types *work* and *education*. Primary activities are defined as activities that take place at one long-term location (e.g. at the workplace). We assume that individuals are required to undertake primary activities for a total daily duration $\tau ^{*}_{\mathcal {P}}$, such that the sum of the duration of each scheduled primary activity is equal to the *primary time budget*: $\sum _{a \in {\mathcal {P}}} \tau _a = \tau _{\mathcal {P}}$. The set ${\mathcal {P}}$ may be empty, if an individual does not need to spend time on any primary activity (i.e. $\tau ^{*}_{\mathcal {P}}$=0).
- *Secondary activity set*: The set ${\mathcal {S}} \subset {\mathcal {A}}$ includes activity types *leisure*, *shopping*, *escort*, *business*, *further training* and *other*. Secondary activities are defined as activities with flexible locations. Hence, they may contain multiple considered locations, one of which is chosen in the optimisation model. In contrast to primary activities, the durations of secondary activities are treated individually rather than their daily sum. This comes with the assumption that people have individual activity duration budgets for secondary activity, but see the duration of primary activities as one daily objective. The set ${\mathcal {S}}$ may be empty, if an individual does not desire to take part at any secondary activity.
- *Schedule*: Each schedule consists of a set of activities ${\mathcal {A}}$. To form a daily schedule, the activities must be in a ordered sequence without overlapping in time, including travel episodes. For this purpose, an individual needs to make the decision about a realised start time *x*, a realised duration $\tau$, as well as a chosen location *l* for each $a \in {\mathcal {A}}$. Locations and sequence of activities result in travel episodes with a mode $\mu$ and a corresponding travel time *tt*. Activities of the same type must not take place consecutively if there is no travel episode in between (i.e. both are at the same location). A schedule always starts with a *dawn* activity at midnight and ends with a *dusk* activity at the defined time period. An example for a schedule is $dawn \rightarrow work \rightarrow leisure\_\textit{2} \rightarrow home \rightarrow leisure\_\textit{1} \rightarrow dusk$. We consider two types of schedules:
- *Realised schedule*: In the choice set of the parameter estimation, it is the empirical schedule as reported in a survey. In the optimisation context, it is the output of the optimisation model.
- *Feasible schedule*: Any possible deviation from a realised schedule can be feasible as long as it fits into the time period constraint and does not contain overlapping activities.
- *Tours*: The set of tours ${\mathcal {T}}$ contains all out-of-home tours in the daily schedule of an individual. Each tour $t \in {\mathcal {T}}$ is defined to contain the subset of activities that take place between two home activities (e.g. $home \rightarrow work \rightarrow shopping \rightarrow home$). The number of tours in a schedule hence equals the number of home activities minus one ($|{\mathcal {T}}| = |{\mathcal {H}}|-1$).
  ${\mathcal {T}}$ includes tour types *work*, *education* and *secondary*. Work and education tours include at least one and at most two primary activities of this type. Tours that include secondary activities only are referred to as secondary tours. The tour type is a static input in the optimisation context of this work.
- *Sub-tours*: A sub-tour is a sequence of secondary activities between two primary activities without any home activity in between (e.g. *work* – *lunch* – *work*).

### Estimation of individual flexibility preferences

This section explains the estimation of activity-specific flexibility parameters. Section "Choice set generation"   gives an insight into the methodology that is used to generate a competitive choice set. The model estimation uses the utility specification as given in section "Utility specification"   and is described in section "Model estimation" 

#### Choice set generation

In discrete choice modelling, individuals evaluate, compare and select alternatives from a choice set of mutually exclusive alternatives (Manski 1977). In many applications, it is assumed that choice sets are fully known by the decision-makers and are fully or partially observed by the modellers. This assumption is unrealistic for applications in which the choice set is large such as location or route modelling.

The way we define the choice set in this paper is similar to the definition proposed by Shocker et al. (1991) in the context of marketing. We model the temporal dimension in the activity-scheduling process as the discrete choice between different combinations of activity timings and durations while also considering travel times between them. We assume that the decision-maker only possesses a partial knowledge of the available opportunities. The set of all available combinations is the *feasible schedule* set. This sample is finite but potentially very large. In addition, the sample of alternatives that the individual actually considers may neither be fully known by the modeller nor readily accessible from traditional data sources.

The utility-based framework described in Pougala et al. (2022) requires the estimation of the parameters of the utility function, namely the penalties for schedule deviations from desired activity start times and durations, and the cost for travelling. The estimation requires a sample of alternatives with a formal description of the sampling protocol. Given the difficulty of this task in this context, we propose a heuristic approach in which choice sets are generated using a combination of the following two methods:

1. *Random alternatives:* We start with generating random schedule alternatives by randomly modifying the activity start times and durations in a given set of activities ${\mathcal {A}}$ as observed in the realised schedule of the MTMC. As the outcomes are random and not based on any empirical distribution, many alternatives are likely to have low levels of utility compared to the observed schedule. Relatively low levels of utility imply that the generated alternatives differ substantially from the observed schedule.
2. *Likely considered alternatives:* We generate activity schedules with variations of activity start times, durations and travel times that are likely to be considered by the decision-maker and hence biased towards high probability schedules. By likely considered activities we mean schedules that are observed by other individuals with similar characteristics (e.g. employment rate, age, urban home, etc.). For this purpose, the sequential activity-based demand model MOBi.plans as presented in Scherr et al. (2020a) is applied to draw distributions from the activities sets in the schedules as reported in the MTMC.

#### Utility specification

As the adapted version of the scheduling framework in this paper focuses on temporal scheduling decisions, it uses the temporal components of the comprehensive utility specification as proposed in Pougala et al. (2022) including travel times that arise from travelling between activities. In our work, the intrinsic attraction of an activity is not modelled with a constant inside the optimisation, but as a discrete choice based on the utilities of all available alternatives prior to the optimisation (see Section "Activity set generation" ). Specifically, we include the utility terms relating to schedule deviations from timing and duration preferences for each activity *a* and the travel times between *a* and the consecutive activity in our optimisation model for the utility specification of schedule alternative *i*:

1 $$\begin{aligned} U_{i} \ = \ \sum _{a \in {\mathcal {A}}_{i}} U_{\text {timing}}(x_a) \ + \ \sum _{a \in {\mathcal {S}}_{i}} U_{\text {duration}}(\tau _a) \ + \ \sum _{O \in \{{\mathcal {P}}_i, {\mathcal {H}}_i\}} U_{\text {duration}}(\textstyle {\sum _{a \in O}\tau _a}) \ + \ \displaystyle \sum _{a \in {\mathcal {A}}_{i} \setminus \{\text {dusk}\}} U_{\text {tt},a}(tt_a) \end{aligned}$$

The components and the associated assumptions are defined as follows:

- $U_{\text {timing}}(x_a)$ indicates the impact for deviating from desired timings for each activity $a \in {\mathcal {A}}_{i}$. The desired start time is defined as $x^{*}_{a}$ and $x_{a}$ is the start time of activity *a* as stated in the choice set for alternative *i*. We introduce the two flexibility parameters $\beta ^{\text {early}}_{a}$ and $\beta ^{\text {late}}_{a}$ which penalise the difference $|x^{*}_{a}-x_{a}|$:
  2 $$\begin{aligned} U_{\text {timing}}(x_a) \ = \ \beta ^{\text {early}}_{a}\max {(0; \ x^{*}_{a}-x_{a})} \ + \ \beta ^{\text {late}}_{a}\max {(0; \ x_{a}-x^{*}_{a})} \end{aligned}$$
- $U_{\text {duration}}(\tau _a)$ captures the impact of deviating from desired durations. In the case of desired duration budgets, we differentiate between home, primary and secondary activities (see definitions in Section "General definitions" ). For primary activities $a \in {\mathcal {P}}$, we compare the sum of all scheduled primary activities (i.e. $\tau _{a} = \sum _{p \in {\mathcal {P}}}\tau _{p}$) to the desired daily duration, or primary time budget ($\tau _{a}^* = \tau _{\mathcal {P}}^*$). The same assumption applies to home activities. For secondary activities $a \in {\mathcal {S}}$, we use the individual desired durations (i.e., $\tau _a^* \ne \tau _b^* \forall a, b \in {\mathcal {S}}, a \ne b$). $\beta ^{\text {short}}_{s}$ and $\beta ^{\text {long}}_{s}$ represent the loss in utility for deviating from a desired duration:
  3 $$\begin{aligned} U_{\text {duration}}(\tau _a) \ = \ \beta ^{\text {short}}_{a}\max {(0; \ \tau ^{*}_{a}-\tau _{a})} \ + \ \beta ^{\text {long}}_{a}\max {(0; \ \tau _{a}-\tau ^{*}_{a})} \end{aligned}$$
- $U_{\text {tt},a}(tt_a)$ is a disutility for the time spent travelling. Since the focus of this work lies on finding flexibility parameters, we fix $\beta _{\text {travel}}$ to be -1. Different travel times for different alternatives are generated using an existing destination and mode choice model (Scherr et al. 2020a):
  4 $$\begin{aligned} U_{\text {tt},a}(tt_a) \ = \ \beta _{\text {travel}} \ tt_{a} \end{aligned}$$

#### Model estimation

Given the proposed utility specification, there are six types of parameters for each activity *a* to be determined: (i) the desired start time $x_a^*$; (ii) the desired duration $\tau _a^*$; and the four penalty terms for deviations from the desired start time and duration (iii) $\beta ^{\text {early}}_{a}$, (iv) $\beta ^{\text {late}}_{a}$, (v) $\beta ^{\text {short}}_{a}$, and (vi) $\beta ^{\text {long}}_{a}$.

We use two different approaches to determine the parameter values for these parameters:

1. For the desired start times and durations ($x_a^*$ and $\tau _a^*$), we use the modal values from empirical distributions for the activity types as found in the MTMC. All activity types are grouped to have uni-modal distributions. Since persons in the MTMC only report on realised start times and durations ($x_a$ and $\tau _a$), this requires the assumption that the desired timings can be approached by the empirical mode of the realised timings.
2. Once the desired start times and durations are defined, the penalty terms ($\beta ^\text {early}_a$, $\beta ^\text {late}_a$, $\beta ^\text {short}_a$, and $\beta ^\text {long}_a$) are estimated from historic schedules using maximum likelihood estimation.

Maximum likelihood estimates $\hat{\beta }$ of the unknown model parameters are given by:

5 $$\begin{aligned} \hat{\beta } = \arg \max _\beta L = \prod _{n\in N}\prod _{i \in C} P_{in}^{y_{in}} \end{aligned}$$

where L is the likelihood function, $P_{in}$ is the choice probability of alternative *i* in the choice set *C*. $y_{in} = 1$ if individual *n* chooses alternative *i* and $y_{in} = 0$ otherwise (Train 2003). Assuming a multinomial logit model, the choice probability for each alternative *i* in the choice set $C_n$ is defined as follows:

6 $$\begin{aligned} P_{in} = \frac{e^{\mu V_{in}}}{\sum _{j\in C_n} e^{\mu V_{jn}}} \end{aligned}$$

with a scale parameter $\mu > 0$.

Note that the maximum likelihood estimation requires an enumeration of the alternatives in the choice set. In the activity-travel context, the full choice set $C_n$ of alternatives is combinatorial. The parameters are therefore estimated on a sample of alternatives $\tilde{C}_n$. The maximisation of the likelihood function yields consistent parameter estimates if the uniform conditioning property is verified: $P_n(i | \tilde{C}_n) = P_n(j | \tilde{C}_n)\ \forall i, j \in \tilde{C}_n$. This is achieved by introducing a correction term $\ln P_n(\tilde{C}_n | i)$ to take into account sampling biases (Ben-Akiva and Lerman 1985):

7 $$\begin{aligned} P_{in} = P_n(i | \tilde{C}_n) = \frac{e^{\mu V_{in} + \ln P_n(\tilde{C}_n | i)}}{\sum _{j\in \tilde{C}_n} e^{\mu V_{jn} \ln P_n(\tilde{C}_n | j)}} \end{aligned}$$

### Schedule optimisation

Having defined the activity-specific flexibility parameters, we now introduce the optimisation model that targets to solve scheduling conflicts in the temporal dimension. Section "Activity set generation" gives an overview over the generated activity sets that are treated as static inputs in the optimisation model. Then,  sections "Optimisation model" and "Tour-based indicators and constraints" explain the implementation of the MILP.

#### Activity set generation

The optimisation framework used in this work needs an activity set ${\mathcal {A}}$ as an input for each individual in a synthetic population (Fig. 1). This activity set ${\mathcal {A}}$ contains the long-term decisions (i.e. primary locations) and non-temporal decisions (i.e. activity participation, number and type of tours, and considered destinations). This requires the assumption that an individual knows the number and type of the activities it desires or needs to participate during the day prior to the time-conflict resolution. In this work, we apply the following sequence of discrete choice models^1^ with the parameters as introduced and explained in more detail in Scherr et al. (2020a):

1. Long-term decisions about primary locations such as a school- or workplace, which depend on the choice of possessing a mobility tool such as a driving license and public transport subscription (Hillel et al. 2020). Additionally, the discrete choice models for the long-term locations consider the attractions of the destination, e.g. the number of jobs in the case of a workplace. This allows for including the location size as a capacity constraint, as demonstrated and validated in Scherr et al. (2020a).
2. Decision about number and type of primary out-of-home tours (i.e. work and education tours) depending on the long-term decisions and their resulting commuting distances as well as number and type of secondary activities within the primary tours. Also, the number and type of sub-tour activities are generated during this choice step.
3. Decision about number of secondary tours as well as the number and type of secondary activities within each secondary tour.
4. Generation of a set of considered locations for each secondary activity based on weighted draws from given distributions. The weights consider location sizes, e.g. large shopping centres have higher attractions compared to a small shop.

Table 1 gives an example of an activity set ${\mathcal {A}}$. The cells with sets in italic letters mean that these decisions are made in the optimisation model. Under consideration of these possible choice options, the activity sequence will be optimised as well. All other cells are static input for the optimisation model. It contains four home activities, which equals three possible out-of-home tours. At this stage, the tours are not fixed in their sequential order. This means that the tour with $id=1$ may also take place at the end of the day and include the dusk activity. The tour with $id=1$ is primary, including one sub-tour activity. Since the sub-tour activity is static, this lunch activity must always take place between the two work activities. However, the two work activities can still swap their sequential order. Also, it will be decided if the accompany activity takes place before or after the work in the optimisation model. Three activities take place within secondary tours ($id=2$ and $id=3$) and cannot be moved to the work tour since the tour type is static. Their sequence is flexible, one shopping activity might end up in the same tour as the leisure activity if it fits best into in time and space preferences. Also, the secondary activities may have multiple considered locations, one of which will be chosen later in the optimisation model.

**Table 1:** Example for an activity set ${\mathcal {A}}$ with the possible decision options

Activity	Tour id	Tour type	Primary activity	Sub-tour activity	Considered locations
Dawn	{*1, 2, 3*}	{*Work, secondary*}	False	False	Home place
Work	1	Work	True	{*True, false*}	Office
Lunch	1	Work	False	True	Office
Work	1	Work	True	{*Tue, false*}	Office
Escort	1	Work	False	False	Kindergarten
Home	{*1, 2, 3*}	{*Work, secondary*}	False	False	Home place
Shopping	{*2, 3*}	Secondary	False	False	{*Shop 1, shop 2*}
Shopping	{*2, 3*}	Secondary	False	False	{*Shop 3, shop 4*}
Home	{*1, 2, 3*}	{*Work, secondary*}	False	False	Home place
Leisure	{*2, 3*}	Secondary	False	False	Gym
Dusk	{*1, 2, 3*}	{*Work, secondary*}	False	False	Home place

The generation of the desired start times and durations for each activity in the context of this case study will be explained later in section "Definition of a desired timing and duration per activity type based on empirical distributions" 

#### Optimisation model

This section summarises our implementation of the MILP, which builds on the theoretical framework introduced by Pougala et al. (2022). Compared to the work of Pougala et al. (2022), we modify the optimisation model in mainly three ways to make it applicable in a real-world case study: first, we simulate the intrinsic attraction of an activity outside of the optimisation model. This allows us to remove the decision variable about activity participation. Second, the choices about activity locations and transport modes are modelled as decision variables for each activity in the activity set and not as separate activities. This is more efficient since we can minimise the size of the initial activity set. Finally, we introduce additional tour-based indicators and constraints to the optimisation model as described in "Tour-based indicators and constraints". Doing so, the search space in the optimisation model is reduced.

For each schedule alternative *i*, an individual has a predefined set of activities ${\mathcal {A}}_i$ containing all activities *a* that are wished or needed to be performed by that individual within a bounded time period $\xi$ (e.g. a 24-h day). In order to maximise the total schedule utility for each alternative *i*, each individual makes the decisions for each activity $a \in {\mathcal {A}}_i$ about: (i) the duration $\tau _a$ and the start time $x_a$, which implies the activity sequence; (ii) the tour id $\vartheta _a$; (iii) the location $\lambda _a$; (iv) the mode $\mu _a$; and (v) the travel time $tt_a$, which is an implication of the sequence, location and mode.

The objective for each individual is to maximise its total schedule utility which is defined in Equation (1):

$$\begin{aligned} \Omega \ = \ \max \ U_{i} (x, \tau , tt) \end{aligned}$$

To this end, we introduce the following decision variables of the optimisation model:

8 $$\begin{aligned} \begin{array}{lcrrrll} x_{a} &{} \in &{} {\mathbb {R}}_+ &{} &{} \forall a \in {\mathcal {A}}_i \\ \tau _{a} &{} \in &{} {\mathbb {R}}_+ &{} &{} \forall a \in {\mathcal {A}}_i \\ tt_{a} &{} \in &{} {\mathbb {R}}_+ &{} &{} \forall a \in {\mathcal {A}}_i \end{array} \end{aligned}$$

where $x_a$ and $\tau _a$ represent start time and duration of activity *a*, respectively, and $tt_a$ represents the travel time from *a* to the following activity.

Another important set of decisions variables are the activity sequence indicators:

9 $$\begin{aligned} \begin{array}{lcrrrll} z_{ab}\in & {} \{0,1\}&\,&\forall a, b \in {\mathcal {A}}_i \end{array} \end{aligned}$$

where $z_{ab} = 1$ is equal to one if activity *a* takes place right before *b*. The travel time from *a* to *b* heavily depends on both trip origin and destination locations as well as the mode used for this trip. For this purpose, we introduce the location and mode choice variables:

10 $$\begin{aligned} \begin{array}{lclllrrll} \lambda _{al} &{} \in &{} \{0,1\} &{} &{} \forall a \in {\mathcal {A}}_i &{} \forall l \in \{1, \dots , |L_{a}|\} &{} =: {\mathcal {L}}_a \\ \mu _{am} &{} \in &{} \{0,1\} &{} &{} \forall a \in {\mathcal {A}}_i &{} \forall m \in \{1, \dots , |M|\} &{} =: {\mathcal {M}} \end{array} \end{aligned}$$

where ${\mathcal {L}}_a$ is the set of all considered locations for activity *a* and *M* is the set of all available modes. The location and mode choice variables have a direct impact on the travel times. The travel time variable can now be set as follows:

11 $$\begin{aligned} \begin{array}{lcllllll} tt_a= & {} \displaystyle \sum _{b \in A, \ l_a \in {\mathcal {L}}_a, \ l_b \in {\mathcal {L}}_b, \ m \in {\mathcal {M}}} \Theta (l_a,l_b, m) \ z_{ab}\lambda _{al_a}\lambda _{bl_b}\mu _{abm}&\,&\forall a \in {\mathcal {A}}_i \end{array} \end{aligned}$$

where the travel time matrix $\Theta$ is exogenous and contains the travel times for all possible combinations of locations and modes within $\bigcup _{a \in {\mathcal {A}}_i} {\mathcal {L}}_a$ and ${\mathcal {M}}$. In the implementation of the optimisation model, we use a linearised form of this multiplication by introducing auxiliary variables.

The optimisation problem implemented in this work is now subject to the constraints:

12 $$\begin{aligned} \sum _{a, b \in {\mathcal {A}}_i} (\tau _a + z_{ab} tt_{a})&\quad = \quad \xi \end{aligned}$$

13 $$\begin{aligned} \delta \ \ \ \le \ \ \ \tau _a&\quad \le \quad \xi&\qquad \forall a \in {\mathcal {A}}_i \end{aligned}$$

14 $$\begin{aligned} z_{ab} \ + \ z_{ba}&\quad \le \quad 1&\qquad \forall a,b \in {\mathcal {A}}_i \end{aligned}$$

15 $$\begin{aligned} z_{a,\text {dawn}} \ \ \ = \ \ \ z_{\text {dusk},a} \ \ \ = \ \ \ \ z_{aa}&\quad = \quad 0&\qquad \forall a \in {\mathcal {A}}_i \end{aligned}$$

16 $$\begin{aligned} z_{ab}&\quad = \quad 0&\qquad \forall a, b \in {\mathcal {P}}_i \ \text {or} \ {\mathcal {H}}_i \end{aligned}$$

17 $$\begin{aligned} \displaystyle \sum _{b \ne a} z_{ab}&\quad = \quad 1&\qquad \forall a \in {\mathcal {A}}_i \setminus \{\text {dusk}\} \end{aligned}$$

18 $$\begin{aligned} \displaystyle \sum _{b \ne a} z_{ba}&\quad = \quad 1&\qquad \forall a \in {\mathcal {A}}_i \setminus \{\text {dawn}\} \end{aligned}$$

19 $$\begin{aligned} \sum _{l \in {\mathcal {L}}_a} \lambda _{al} \ \ \ = \ \ \ \sum _{m \in {\mathcal {M}}} \mu _{am}&\quad = \quad 1&\qquad \forall a \in {\mathcal {A}}_i \end{aligned}$$

20 $$\begin{aligned} (z_{ab}-1) \xi \ \ \le \ \ x_a \ + \tau _a \ + \ z_{ab} tt_{a} \ - \ x_b&\quad \le \quad (1-z_{ab}) \xi&\qquad \forall a,b \in {\mathcal {A}}_i \end{aligned}$$

21 $$\begin{aligned} \gamma _a^{-} \ \ \ \le \ \ \ x_{a}&\quad \le \quad \gamma _a^{+} - \tau _a&\qquad \forall a \in {\mathcal {A}}_i \end{aligned}$$

Constraint (12) ensures that the total sum of all activity durations and travel times is exactly equal to the predefined time period $\xi$ the schedule should fill out. The next constraint (13) guarantees that each duration is between the minimal duration $\delta$ and maximal duration $\xi$. Constraint (14) imposes that an activity takes place either before or after another activity, possibly neither but never both. The bundled constraint (15) assures that no activity takes place before dawn or after dusk and that an activity cannot follow itself. Constraint (16) enforces that two primary activities or home activities cannot take place consecutively since this would break the general schedule structure as described in section "General definitions". The two constraints (17) and (18) ensure that every activity is preceded by another activity (except for dawn) and is also followed by some other activity (except for dusk). To ensure that only one location and mode is chosen per activity, constraint (19) is set in place. Constraint (20) makes sure that – if activity *b* follows activity *a* – the start time of *a* plus duration plus the travel time from *a* to *b* is exactly equal to the start time of *b*. The last constraint (21) guarantees that every activity can only take place within its designated feasible time window (e.g., opening hours).

#### Tour-based indicators and constraints

We extend the optimisation model by several indicator variables and constraints that capture a general schedule structure (e.g. number of primary activities per tour). These indicator variables are later used to constrain the decision options for certain variables such as tour type or sub-tour activity to the sets as introduced in Table 1.

The first introduced tour-related variable is a tour indicator:

22 $$\begin{aligned} \begin{array}{lcrrrllllll} \vartheta _{at}\in & {} \{0,1\}&\,&\forall a \in {\mathcal {A}}_i&\forall t \in {\mathcal {T}}_i \end{array} \end{aligned}$$

where ${\mathcal {T}}_i$ is the set of all possible out-of-home tours that can occur in a given schedule alternative *i*. If activity *a* takes place in tour *t*, $\vartheta _{at}$ equals one. The corresponding tour constraints are:

23 $$\begin{aligned} \begin{array}{lcrrrll} \vartheta _{at} &{} \le &{} \vartheta _{bt} - z_{ab} + 1 &{} &{} \forall a \in {\mathcal {A}}_i \setminus \{\text {dusk}\}&{} \forall b \in {\mathcal {A}}_i \setminus {\mathcal {H}}_i &{} \forall t \in {\mathcal {T}}_i\\ \vartheta _{at} &{} \ge &{} \vartheta _{bt} + z_{ab} - 1 &{} &{} \forall a \in {\mathcal {A}}_i \setminus \{\text {dusk}\} &{} \forall b \in {\mathcal {A}}_i \setminus {\mathcal {H}}_i &{} \forall t \in {\mathcal {T}}_i \end{array} \end{aligned}$$

where ${\mathcal {H}}_i$ is the set of all home activities. If $z_{ab} = 1$, the two constraints make sure that $\vartheta _{at} = \vartheta _{bt}$. Or in other words, if activities *a* and *b* are executed in sequence, both must be in the same tour. An exception is made for home activities, as these functions are break points between out-of-home tours. The break points make sure that the tour indicator $\vartheta _{at}$ always changes at a home activity *h*.

We use this tour-indicator variable to assign a tour type to every tour, which is either *work*, *education* or *secondary* (see section "General definitions"). The type of the tour depends on the presence of a primary activity of a certain type within that tour. For the scope of this work, we keep the tour type fixed for each activity. Therefore, we add constraints that ensure that every activity *a* takes place in a tour with a type corresponding to the predefined activity tour type:

24 $$\begin{aligned} \begin{array}{lcrrrll} \displaystyle \sum _{t \in {\mathcal {T}}_i(a)} \vartheta _{at}\ge & {} 1&\,&\forall a \in {\mathcal {A}}_i \end{array} \end{aligned}$$

where ${\mathcal {T}}_i(a)$ is the set of tours with type equal to the tour type of activity *a*. With this constraint, it is ensured that *a* takes place in at least one tour, and this tour has the type as defined in the activity set ${\mathcal {A}}_i$.

Furthermore, we use the tour indicator to fix the number of primary activities per tour. This is achieved with the following constraint:

25 $$\begin{aligned} \begin{array}{lcrrrll} \displaystyle \sum _{a \in {\mathcal {P}}_i} \vartheta _{at}= & {} p_t&\,&\forall t \in {\mathcal {T}}_i \end{array} \end{aligned}$$

where $p_t$ is the number of primary activities in tour *t* as predefined in the activity set ${\mathcal {A}}_i$.

To add modelling capability depending on the position of activity *a* in the schedule, we next introduce a variable that functions as a sub-tour indicator:

26 $$\begin{aligned} \begin{array}{lcrrrllllll} \psi _{a}\in & {} \{0, 1\}&\,&\forall a \in {\mathcal {A}}_i \end{array} \end{aligned}$$

together with the following constraints

27 $$\begin{aligned} \begin{array}{lcrrrll} \psi _{a} &{} \le &{} \psi _{b} - z_{ab} + 1 &{} &{} \forall a \in {\mathcal {A}}_i &{} \forall b \in {\mathcal {A}}_i \setminus {\mathcal {P}}_i\\ \psi _{a} &{} \ge &{} \psi _{b} + z_{ab} - 1 &{} &{} \forall a \in {\mathcal {A}}_i &{} \forall b \in {\mathcal {A}}_i \setminus {\mathcal {P}}_i \end{array} \end{aligned}$$

where ${\mathcal {P}}_i$ is the set of all primary activities. Per definition, a sub-tour is not allowed to start or end at home. For this reason, we fix the sub-tour indicator for activities in the set ${\mathcal {H}}_i$ to be zero:

28 $$\begin{aligned} \begin{array}{lcrrrllllll} \psi _{a}= & {} 0&\,&\forall a \in {\mathcal {H}}_i \end{array} \end{aligned}$$

Sub-tours are constructed in a way that the first instance of the primary activity is part of the sub-tour. The second instance after the sub-tour is not part of the sub-tour anymore, since the following trip is going home-wards (possibly with another activity in between). We use the information about the number of primary activities in a tour $p_t$ as given in constraint (25) to introduce a minimum a amount of sub-tour activities:

29 $$\begin{aligned} \begin{array}{lcrrrllllll} p_t\le & {} \sum _{a \in P_t} \psi _{a} \ + 1&\,&\forall t \in {\mathcal {T}}_i \end{array} \end{aligned}$$

which means that $\psi _{a}$ has to be at least one for primary activities in tour *t* if two primary activities are present ($p_t=2$). This will always be the first instance of the primary activities since the second instance is directed towards a home activity at some point and hence forced to be zero because of constraint (28). Lastly, at least one secondary sub-tour activity must happen between them since the two primary activities are not allowed to take place directly after each other (16).

## Case study

This section presents the city-scale application of the proposed framework to the full-time workers in a synthetic population on a city-scale for Lausanne, Switzerland. In  section"Overview", we give a general overview of the case study with the external inputs and general assumptions. In section "Definition of a desired timing and duration per activity type based on empirical distributions"  we defines the desired timings and durations for all activity types which are used for both parameter estimation and schedule optimisation based on empirical data. In section "Flexibility parameter estimation", the estimation of the flexibility parameters is demonstrated and discussed. Finally, section "Optimised schedules" gives an overview of the implementation of the optimisation model and shows the results of applying the flexibility parameters to a synthetic population using our mathematical model.

### Overview

The proposed framework is applied to a synthetic population of full-time workers living in the city of Lausanne, Switzerland. With more than 140’000 inhabitants (as of 2017), it is one of the biggest cities in Switzerland. Since behaviour depends on specific person attributes – for example, a student is likely to have a different behaviour compared to a full-time worker – the population must be divided into different groups and the flexibility parameters calibrated separately for each group. For demonstration purposes in this work, we focus on the estimation of the parameters for full-time workers (employment rate higher than 80 % and not primarily in education). As shown in Fig. 1, the framework needs two static inputs:

1. *Reported schedules*: We use the reported schedules from the Swiss Mobility and Transport Microcensus (MTMC) (BfS and ARE 2017). The computer-assisted telephone survey takes place every 5 years, most recently in 2015. It contains a sample of 57’090 persons from all over Switzerland. Each person reports on their conducted mobility schedule during a full day. A cleaning procedure is applied to remove persons who have a reporting date on the weekend and persons with non-valid schedules. Non-valid schedules include schedules that are not fully reported and schedules which do not comply with the constraints of the model (e.g. a person must always return home in the evening or primary and home activities must not take place consecutively). After the cleaning procedure, around 40’000 reported schedules remain in the observation set. For full-time workers in the whole country of Switzerland, the cleaned set in the MTMC contains 10’110 reported schedules.
2. *Synthetic population*: The nation-wide synthetic population of Switzerland for the year 2017 is generated by Bodenmann et al. (2019). It contains a very detailed person database with attributes such as age, employment rate, etc. as well as household structures and residences. In the synthetic population, almost 50’000 full-time workers are present in the city of Lausanne.

An additional third input for the model are travel times between different locations for all modes (see section "Optimisation model"). We use static travel times which are derived from a network assignment using MATSim (Horni et al. 2016) and the Switzerland scenario from Scherr et al. (2020a). As a simplified assumption, we use constant travel times over the day. The public transport travel times are averaged over one hour (i.e. 7:00-8:00 h). Since the service frequency and the travel times of public transport in Switzerland are fairly constant throughout the day, constant travel times over the day are assumed to be valid. Car travel times are defined as the maximum between the congested state of the morning peak and the evening peak, which is a rather pessimistic assumption for car traffic. Travel times for the modes walk and bike are calculated based on the beeline distance between two locations, a detour factor and an average speed (walk: 4.7 km/h; bike: 13.0 km/h as reported in BfS and ARE (2017)).

For both choice set generation and schedule optimisation, the following model steps are generated based on existing discrete choice models (see section "Activity set generation") as proposed by Scherr et al. (2020a) and assumed to be given in this case study: (i) the set of tours ${\mathcal {T}}$; (ii) the tour type of each activity $\vartheta _{at} \ \forall t \in {\mathcal {T}} \ \forall a \in {\mathcal {A}}$; (iii) the number of home activities $|{\mathcal {H}}|=|{\mathcal {T}}|+1$; (iv) the set of primary activities ${\mathcal {P}}$ with a type and a number of primary activities per tour $p_t$; (v) the set of secondary activities ${\mathcal {S}}$ with a given activity type; (vi) the set of secondary sub-tour activities $\psi _a=1$; and (vii) the considered destinations ${\mathcal {L}}_{a} \ \forall a \in {\mathcal {A}}$.

### Definition of a desired timing and duration per activity type based on empirical distributions

A crucial element of the proposed framework is the definition of activity types for a specific person group with corresponding desired timings $x^{*}_{a}$ and durations $\tau ^{*}_{a}$ for each activity $a \in {\mathcal {A}}$. As these terms are defined in the utility specification in Equation (1), they are a common requirement for both parameter estimation and schedule optimisation. We use the main activity types for home, primary and secondary activities as defined in section "General definitions". However, the flexibility of activity start times and durations might vary throughout the day (e.g. the flexibility of starting with a work activity might be different in the morning compared to starting with the afternoon work activity after lunch). Therefore, we split the main activity types into different behaviourally homogeneous sub-activities. By behaviourally homogeneous, we mean activities that have uni-modal distributions in the dimensions of both $x^{*}_{a}$ and $\tau ^{*}_{a}$ in the reported schedules. This is based on the assumption that activities with reported uni-modal distributions actually belong together and have the same meaning in terms of flexibility for specific desired timings and durations across all the individuals in a specific person group. As explained in section "Model estimation", $x^{*}_{a}$ and $\tau ^{*}_{a}$ are then defined based on the modal values from empirical distributions in the MTMC. To find uni-modal distributions, the available information in the activity set ${\mathcal {A}}$ is used (e.g. number of primary activities or the number and type of tours).

Table 2 shows the empirical analysis of the activity types as observed in the MTMC. We divide activities into sub-activities by applying a manual cluster analysis based on visually observing the distributions for activity timings and durations. Our goal is to find uni-modal distributions while not splitting the activity type into too small clusters. In this work, all observations are assumed to be normally distributed. For each sub-activity distribution, we derive the normal distribution from the empirical distribution in the MTMC including the modal value and the standard deviation for both $x^{*}_{a}$ and $\tau ^{*}_{a}$.

**Table 2:** Empirical analysis including desired timings and durations for activity types of full-time workers in the MTMC. Total number of observed schedules is 10’110

Activity	Sub-activity	Description	Avg. occurrence	Des. timing $x^{*}$ [h]		Des. duration $\tau ^{*}$ [h]	
	Indicator		Per schedule	mode	std	mode	std
Work	$p_{1}$	First in set	0.80	7.4	0.9		
	$p_{2}, ..., p_{n}$	Following	0.31	13.2	0.3		
	$\sum _{a \in {\mathcal {P}}}{\tau _{a}}$	Duration budget				9.5	0.5
Leisure	$\psi =1$	Lunch	0.10	12.1	0.1	0.9	0.1
	$\vartheta _{work}=1$	Work tour	0.11	18.0	0.5	0.8	0.3
	$\vartheta _{secondary}=1$	Secondary tour	0.17	19.2	0.8	1.8	0.6
	$|{\mathcal {P}}|=0$	No primary activity	0.16	13.0	4.0	1.5	0.7
Shopping	$\psi =1$	Between work	0.06	12.1	0.1	0.4	0.2
	$\vartheta _{work}=1$	Work tour	0.16	17.2	0.5	0.4	0.2
	$\vartheta _{secondary}=1$	Secondary tour	0.22	14.0	1.5	0.4	0.2
Escort	$x\le$ 12:00 h	Drop off	0.08	7.5	0.3	0.3	0.1
	$x>$ 12:00 h	Pick up	0.08	18.0	0.3	0.3	0.1
Business		All day	0.03	10.0	1.0	4.0	2.0
Education		All day	0.02	18.0	2.0	3.5	0.5
Other		All day	0.19	10.0	1.0	4.0	2.0
Dawn			1.00	0.0			
Dusk	$|{\mathcal {T}}|>1$	Multi tours	0.41	15.4	1.0		
	$|{\mathcal {T}}_{work}|=1$	One work tour	0.12	18.0	0.5		
	$|{\mathcal {T}}_{secondary}|=1$	One secondary tour	0.47	16.4	0.7		
Home	$h_{1} \wedge |{\mathcal {T}}_{work}|=2$	Lunch	0.14	12.0	0.1		
	$|{\mathcal {T}}_{work}|>0$	Work tour present	0.25	17.2	0.2		
	$|{\mathcal {T}}_{work}|=0$	No work tour	0.10	17.3	0.4		
	$\sum _{a \in {\mathcal {H}}}{\tau _{a}}$	Duration budget				12.9	2.0

A straightforward example is the activity type *work* in Table 2. It is divided into the two clusters of the first occurrence in the activity set ${\mathcal {A}}$ and – if present – following occurrences of work activities in ${\mathcal {A}}$. Our analysis shows that ${\mathcal {A}}$ contains at least one work activity in 80 % of the schedules (average occurrence 0.80). This means that on an average work day in Switzerland, 80 % of all full-time workers go to their work place at least once. In that case, workers are observed to begin their work activity with a modal timing of about 7:30 h. The observed distribution has a standard deviation of 0.9. The average occurrence of having more than one work activity in the activity set is 0.31, meaning that 31 % of the all individuals participate in at least one activity between the two work activities (e.g. returning home, or going out for lunch). The desired timing $x^{*}_{a}$ for returning back to the work place is at 13:15 h and follows more pronounced peak (standard deviation of 0.3). The total duration budget $\tau ^{*}_{a}$ (sum over all work activity durations in ${\mathcal {A}}$) for full-time workers is distributed around a modal value of 9.5 hours as observed in the survey.

Another notable example is the activity *home*. In the case of two work tours ($|{\mathcal {T}}_{work}|=2$), exactly one home activity must take place between the two out-of-home tours per definition. This home activity between two work tours has a modal start time at 12:00 h and shows a very strong peak. Additionally, full-time workers are returning back home in average between 17:00 and 18:00 h in the case of at least one work activity in ${\mathcal {A}}$. The analysis indicates that there is a desire to be back home at around 18:00 h. The observed total home duration budget is distributed around a mode of around 13 h (sum over all home activity durations in ${\mathcal {A}}$), meaning that the group of the full-time workers in average spends more than half of the day at home.

### Flexibility parameter estimation

Besides the desired timings $x^{*}_{a}$ and duration $\tau ^{*}_{a}$ for each activity type, the estimation of flexibility parameters relies on strategic choice sets containing alternatives that decision-makers are likely to consider, as explained in section "Choice set generation". In this case study, a choice set is generated consisting of the realised schedule as well as a sample of 100 *random alternatives* and 100 *likely considered alternatives*. The alternative schedules are generated based on the realised schedules of all full-time workers as reported in the MTMC under consideration of the general schedule structure as explained in section "Activity set generation". The choice set size has been chosen such that computational time of both choice generation and parameter estimation remain manageable (i.e. under one day). A sensitivity analysis to investigate the impact of the size must be conducted in future work.

Having generated a choice set and defined the desired timings $x^{*}_{a}$ and durations $\tau ^{*}_{a}$ for each activity type (see Table 2),we estimate the flexibility parameters $\beta ^{early}_{a}$, $\beta ^{late}_{a}$, $\beta ^{short}_{a}$, and $\beta ^{long}_{a}$ of Equation (1). The aim of the flexibility parameters is to express behavioural preferences when it comes to resolving time conflicts between different types of activities. The parameters penalise deviations from desired timings and duration for each activity $a \in {\mathcal {A}}$ depending on the type of the activity. The higher the penalty compared to another activity, the less flexible or the more rigid the individual is when deviating from its desired timing or duration. In this case, it is more likely to reschedule another activity in a conflicting situation. To estimate these parameters, we apply the estimation routine as explained in section "Model estimation"  and use the software PandasBiogeme (Bierlaire 2020).

Table 3 shows the resulting flexibility parameters after applying the proposed estimation routine to the generated choice set. Empty cells mean that these parameters are not part of the utility specification. Cells with a $\beta$ that equals 0 are not significant at a 5% level. In terms of being earlier or later than the desired timing $x^{*}$, the lunch activities (either at home or in a restaurant) show the highest penalties (or loss in utility). This means that people are less willing to deviate from the desired timing for lunch activities compared to other activity types and try to move other activity types in the case of a conflict. Therefore, the desired timing for lunch activities (around 12:00 h as shown in Table 2) is a rigid point in time in the daily schedule of a full-time worker. The parameter for being early ($\beta ^{early}$) at lunch is penalised by a factor of around two compared to being late ($\beta ^{late}$) at lunch. This means that full-time workers weight – in terms of utility – being early at lunch by 5 min equal to being late by 10 min.

**Table 3:** Flexibility parameter values and summary statistics for full-time workers. All parameters are significant at 5.0 % level

Activity type	Parameter values			
	$\beta ^{early}_{a}$	$\beta ^{late}_{a}$	$\beta ^{short}_{a}$	$\beta ^{long}_{a}$
Work:first in set	− 0.518	− 0.401		
Work:following	− 0.317	0		
Work:duration budget			−0.022	0
Leisure:lunch	−1.587	− 0.760	−7.614	−1.314
Leisure:work tour	− 0.189	0	−0.842	−0.085
Leisure:secondary tour	− 0.058	0	− 3.087	−0.693
Leisure:no primary activity	− 0.096	0	−1.505	−0.649
Shopping:between work	− 0.623	−0.472	0	−2.279
Shopping:work tour	− 0.143	− 0.280	0	−0.387
Shopping:secondary tour	− 0.229	− 0.468	− 6.003	−0.746
Escort:drop off	− 1.106	0	0	− 4.171
Escort:pick up	− 0.152	0	0	−0.651
Business:all day	− 0.182	− 0.263	−0.345	−0.884
Education:all day	− 0.281	− 0.405	−0.361	−0.913
Other:all day	− 0.737	− 0.530	0	0
Dawn	0	0		
Dusk:multi tours	− 0.662	0		
Dusk:one work tour	0	0		
Dusk:one secondary tour	0	− 0.047		
Home:lunch	−2.189	− 1.067		
Home:work tour present	−0.072	− 0.397		
Home:no work tour	−0.048	0		
Home:duration budget			0	− 0.373
*Summary statistics*				
Number of parameters	68			
Sample size	10’110			
Initial log-likelihood	−57’443.43			
Final log-likelihood	−48’548.23			
$\bar{\rho }^2$	0.154			
Estimation time (h)	12:20			

Another interesting result is that full-time workers get a relatively high penalty if they do not start their first work activity at the desired time (around 7:30 h). They are not flexible with starting the first work activity, which highly affects the timing when people leave their homes in the morning. The home activity shows a high penalty for being late in the case of a work activity being present in the activity set. Hence, workers are also not flexible with returning back home in the evening when they have to work on that day. The flexibility parameters confirm that it is important for persons to be back home at a specific point in time (around 18:00 h), for example to have dinner with the other household members. Being more rigid with both starting and ending work activities compared to other activity types like leisure results in a constraint time frame for work activities between the desired timing of the first work activity and the desired timing of the returning-home activity. The secondary activities show in general lower penalties (like *leisure* or *shopping*) and are more likely to deviate from the desired timing, with some exceptions like the activity types *escort* in the morning or *other* that show higher penalties and therefore have more rigid timings.

In terms of deviating from the desired duration $\tau ^{*}$ of an activity, some activity types show strong penalties $\beta ^{late}$ and $\beta ^{short}$. For example, the full-time workers get a high penalty $\beta ^{short}$ for a short lunch activity. As a result, they are not willing to take a lunch break shorter than the desired duration (0.9 h, see Table 2). Also, it is an interesting result that full-time workers do only apply a small penalisation to a short work duration and do not penalise a long work duration. A possible interpretation is that they are in general flexible with their work duration, but the duration is determined by other factors like specific office hours. The flexibility parameter for a longer total time spent at home compared to the desired duration (12.9 h) indicates that the group of the full-time workers penalise being too long at home during the day. This means that they actively have the need or the desire to participate in activities outside of the home for a certain time (around half) of the day.

### Optimised schedules

For the simulation of the schedules in this case study, we implement the MILP as described in sections "Optimisation model" and "Tour-based indicators and constraints" and in the open-source optimisation framework OR-Tools^2^ for Python. This general-purpose library allows for applying multiple solvers. The open-source SCIP-solver (Achterberg et al. 2008) is employed using default settings. These settings include a relative optimality gap of 0%, which guarantees that the solution is optimal. The SCIP-solver employs a mix of Branch-and-Bound (Lawler and Wood 1966) and Cutting-Plane (Marchand et al. 2002) generation techniques. Additionally, the Ray^3^ library is implemented to provide an efficient way for parallel computing. The simulation greatly benefits from parallel computing since we can – assuming a computer with an infinite number of cores – parallelise the simulation by the number of individuals. The computer used for the simulation is a physical server with 48 cores and the operating system is Microsoft Windows Server. Using 42 cores, 50k schedules are optimised in around 1 hour. The computation time scales linearly by the amount of cores, which results in an average processing time of 3 seconds per schedule and core.

With this implementation of the optimisation model as well as all the inputs including flexibility parameters from Table 3, the framework is applied to each full-time worker as of the synthetic population of Lausanne, Switzerland. Firstly, we generate an activity set ${\mathcal {A}}$ for each individual, as described in section "Model estimation". For each activity $a \in {\mathcal {A}}$, we then take a random draw from a normal distribution to assign a desired timing $x^{*}_{a}$ and duration $\tau ^{*}_{a}$ based on its activity type as given in Table 2. The output of the optimisation model is a realised schedule with a final choice for each decision variable as given in section "Optimisation model" for the individual of the synthetic population.

A minority of the full-time workers (around 7 %) makes the decision of not participating in any activity outside of the home during the day ($|{\mathcal {A}}|=0$). After removing them from the optimisation model, around 45'000 schedules are simulated. In the optimisation model, the individuals may choose among the transport modes *walk*, *bike*, *public transport* (buses, trains, and all other means) and *car as a passenger*. If the individual owns a driving license and there is a car available in the household, the mode alternative *car as a driver* is also listed in the set of modes ${\mathcal {M}}$. The mode has to remain the same throughout an out-of-home tour. The time period $\xi$ is 24 hours, meaning that every person must be back home at midnight. The feasible time window $\gamma _a^{-}$ and $\gamma _a^{+}$ for each activity type is 4:00 h to 23:00 h, respectively. The only exception is the activity type leisure, with a $\gamma _a^{+}$ of 23:50 h.

**Fig. 2:**
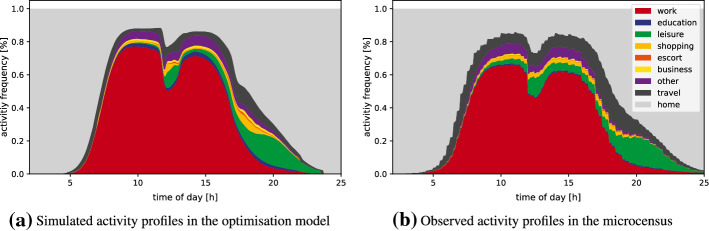
Simulated activity profiles of the simulation compared to the Swiss microcensus

Figure 2 depicts the final aggregated results of the application of the proposed framework to each full-time worker in Lausanne. It shows the relative frequency of individuals who are participating at a certain activity type at a given time of the day (i.e. activity profiles). The figure compares the results of the framework (left Fig. 2a) to the empirically observed activity profiles in the MTMC (right Fig . 2b). Overall, the results of our framework fit the curve of the MTMC nicely. Everyone starts the day at home (light grey area). Between 7:00 h and 17:00 h, the work activity (red) is dominating. The work activity shows a steep peak in the morning and at 10:00 h, between 60 and 80 % of all full-time workers are staying at their work place. At 12:00 h, there is a sudden discontinuity, when people are going to have lunch. This is also indicated by an increasing participation in lunch/leisure (green) and home activities. In the evening, the end time of work activities follows a less steep distribution compared to the morning. After 19:00 h, the dominated activity (besides home) is leisure, which typically ends between 21:00 h and 22:00 h. Only few persons in MTMC are participating at leisure activities after 24:00 h, which is not captured by the model since we constrain the time period to be 24 hours.

In general, travelling (dark grey) consumes a small percentage of the day of a full-time worker. But steep transitions from one activity type to another mean that a lot of persons are travelling during that time period, which causes the traffic peaks. Compared to the profile in the MTMC, the model tends to underestimate the travel episodes. This can be explained by the simplified assumption of a constant travel penalty $\beta _{travel}=-1$ (see section "Model estimation") across all modes of transport. Due to this assumption, the individuals are just minimising travel time in the optimisation model and do not consider mode choice aspects such as costs or personal preferences.

**Fig. 3:**
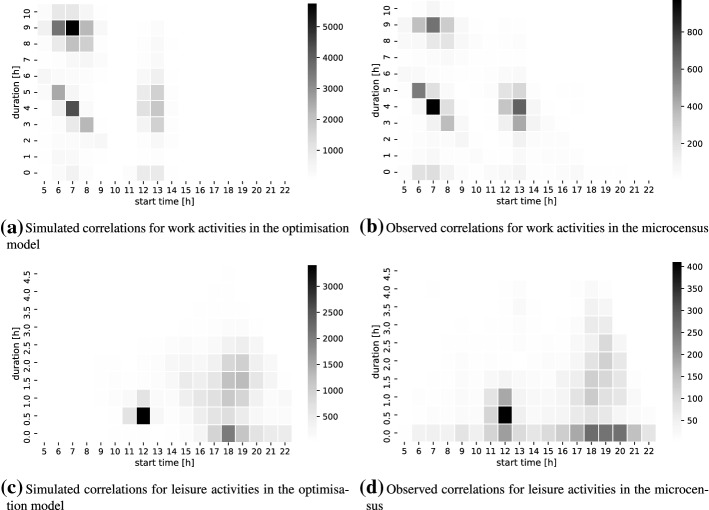
Simulated correlation between start times and durations for selected activity types compared to the Swiss microcensus

Another aim of the proposed framework is to capture the interactions in the temporal dimension. The temporal dimension mainly includes the choices of activity start times and durations. Travel episodes play a minor role in average as shown in Fig. 2. Figure 3 demonstrates the correlation of start times and durations for work and leisure activities. The darker the area, the higher the number of observations that are made within this time frame. The figure compares the realised schedules of the optimisation model (left) to the observed schedules in the MTMC (right). Again, there is an excellent fit between the simulation and the empirical observation. Work activities (Fig. 3a and 3b) show two different types of behaviour: (i) the work activities which are starting early in the morning and take place for around 9 hours; and (ii) the ones which are divided into two separate activities starting in the morning for 4 hours and than starting again in the afternoon for another 4 hours. The model slightly overestimates the number of persons who are working for the full 9 hours.

The leisure activities (Figs. 3c and 3d) are also captured well by the model. The distribution shows a peak at lunch time for 30 to 60 minutes. In the evening, both short and longer leisure activities are taking place, and both types of leisure activities are well represented in the model. During the day, the model underestimates the participation at leisure activities of short duration (i.e. under 30 minutes).

## Discussion

The presented work proposes a framework with a deep behavioural realism for the temporal scheduling decisions in activity-based modelling. Using this framework, time-of-day relevant studies can be modelled. One example is the global COVID-19 pandemic, which may cause a higher prevalence in working from home. The increased flexibility in work-scheduling decisions likely influence how people organise their day. It will be interesting to investigate how sensitive the proposed framework responds to questions such as (i) do people extend the time spent at home? (ii) do they travel longer distances to the work place? (iii) do they extend the duration of their leisure activities? The answers may help practitioners to understand mobility behaviour and to plan future infrastructure, e.g. with regards to the peak hour problem.

To achieve this, the framework must be coupled with a traffic assignment model. At the moment, it cannot predict network loads, e.g. the number of rail passengers boarding a train at a certain time. For this purpose, the optimised schedules must be fed into a traffic assignment, e.g. using the software MATSim (Horni et al. 2016). With the results of the traffic assignment, transport planners can assess policy studies and the need for future network capacity based on network loads.

Additionally, network loads per transport mode are needed. The focus of this work lies on activity timings and not on estimating a mode choice model. A mode choice model would require an extension of the estimation routine to find mode choice parameters that express behavioural preferences and that quantify the influence of level of service indicators (e.g. parking costs, travel time or service frequency). Also, constant travel times are assumed over a 24h-day for all modes of transportation. Particularly the car travel times are depending on the time of the day as travel times differ a lot between peak and off-peak times. For this reason, time-of-day dependent travel times would be an enhancement of this framework when focusing on mode choice.

Household interactions are another relevant modelling aspect that goes beyond the scope of this work. In the current work, we optimise the daily schedules for each individual separately. However, it must be assumed that in reality, many individual decisions are influenced by the decisions of other household members (Gupta and Vovsha 2013). Introducing household constraints or maximising the utility over an entire household is an important avenue for future research in order to achieve more behavioural realism.

The main objective of this work is implementing a working and feasible solution to the activity-based scheduling problem with a general utility specification. To express the problem more comprehensively, further research needs to investigate more advanced utility specifications. For example, all flexibility parameters in this work are defined to be linear penalties. Balać et al. (2018) suggest a non-linear utility function to score the activities in a schedule. It would be interesting to investigate if non-linear (or piece-wise linear) penalties are able to improve the model statistics. Particularly piece-wise linear penalties would be a desirable extension of the utility specification. They would allow for adding an extra penalty to large deviations from desired timings compared to smaller deviations.

Within the optimisation model, the decisions about activity locations are only made for secondary activities such as leisure or shopping. The long-term locations for primary activities (e.g. the workplace) are simulated outside of the optimisation using existing choice models from Scherr et al. (2020a) and treated as static input. Also, the attraction of a location (e.g. the number of jobs) is neglected in the proposed utility specification. For a better representation of the size or the capacity of a location as well as for simulating the decision about long-term locations, the utility specification should be extended by an attraction factor for each individual location.

In the presented case study, the optimisation model constrains opening and closing times for each activity type, but the data is not collected for each specific location. Opening times play an important role in daily scheduling decisions as they are hard constraints. Future work should address this limitation by collecting data for location-related opening times and simulating it within the optimisation model.

We assume that people have preferences for activity start times and durations, but not for activity end times. In reality, it might be the end time that is significant for certain activity types. It goes beyond this work to justify which timing is important for each individual and each activity. Future research should investigate the mentioned behaviour in more detail to find the most relevant variable for each activity.

Additionally, our approach uses fixed desired activity timings and durations, which are found by statistical and visual investigation of the empirically reported schedules (modal values). In the future, a possible improvement of the estimation routine is implementing a mixed logit model (Ben-Akiva et al. 2001) to estimate distributed values for the desired timings and durations.

Lastly, we introduce the heuristic method of combining an existing activity-based model with a random alternative generator to generate the choice set for the parameter estimation. This relies on already having a fully operational activity-based model. Since this is often not the case, a choice set needs to be generated from scratch containing alternatives that a decision-maker is likely to have considered for a reported schedule. A possible direction to design a choice set from scratch is to apply a Metropolis Hastings algorithm (Pougala et al. 2021; Yamamoto et al. 2001).

## Conclusion

In this work, we demonstrate the capabilities of the presented framework to solve the activity-based scheduling problem and to reproduce empirical observations from the Swiss MTMC (BfS and ARE 2017). Methodologically, this is accomplished by combining two modelling components: (i) an estimation routine to quantify individual flexibility parameters based on maximum likelihood estimation; and (ii) a simulation approach that uses the flexibility parameters to resolve temporal conflicts (i.e. activity timings and durations) in order to maximise the schedule utility for each individual as part of a synthetic population. The simulation of the temporal choice dimensions is implemented as a MILP and extends the work of Pougala et al. (2022). For the non-temporal choice dimensions (i.e. activity participation, tour bundling, and considered locations), we simulate a sequence of traditional discrete choice models as introduced by Scherr et al. (2020a).

Our proposed method provides a holistic representation of the scheduling process and uses continuous temporal variables within a utility-maximisation framework. It allows for behavioural realism especially in the temporal dimension, which is often modelled based on simplified assumptions in the literature (Bhat et al. 2004; Auld et al. 2009; Saadi et al. 2016). The method is demonstrated to estimate flexibility parameters that produce close-to-reality results in a real-world application on a city level.

## Footnotes

1 The discrete choice models include socio-demographic attributes as well as level of service indicators (e.g. accessibility or parking costs).

2 https://developers.google.com/optimization, last accessed: 29.08.2021

3 https://ray.io/, last accessed: 29.08.2021
